# Chitin-Glucan Complex Hydrogels: Physical-Chemical Characterization, Stability, In Vitro Drug Permeation, and Biological Assessment in Primary Cells

**DOI:** 10.3390/polym15040791

**Published:** 2023-02-04

**Authors:** Diana Araújo, Thomas Rodrigues, Catarina Roma-Rodrigues, Vítor D. Alves, Alexandra R. Fernandes, Filomena Freitas

**Affiliations:** 1 Associate Laboratory i4HB—Institute for Health and Bioeconomy, School of Science and Technology, NOVA University Lisbon, 2829-516 Caparica, Portugal; 2 UCIBIO—Applied Molecular Biosciences Unit, Department of Chemistry, School of Science and Technology, NOVA University Lisbon, 2829-516 Caparica, Portugal; 3 UCIBIO—Applied Molecular Biosciences Unit, Departmento Ciências da Vida, NOVA School of Science and Technology, 2829-516 Caparica, Portugal; 4 LEAF—Linking Landscape, Environment, Agriculture and Food, Associate Laboratory TERRA, Instituto Superior de Agronomia, Universidade de Lisboa, Tapada da Ajuda, 1349-017 Lisboa, Portugal

**Keywords:** hydrogels, chitin-glucan complex, polymer concentration, drug delivery, Franz diffusion cell, permeation studies

## Abstract

Chitin-glucan complex (CGC) hydrogels were fabricated by coagulation of the biopolymer from an aqueous alkaline solution, and their morphology, swelling behavior, mechanical, rheological, and biological properties were studied. In addition, their in vitro drug loading/release ability and permeation through mimic-skin artificial membranes (Strat-M) were assessed. The CGC hydrogels prepared from 4 and 6 wt% CGC suspensions (Na5_1_*^4^ and Na5_1_*^6^ hydrogels, respectively) had polymer contents of 2.40 ± 0.15 and 3.09 ± 0.22 wt%, respectively, and displayed a highly porous microstructure, characterized by compressive moduli of 39.36 and 47.30 kPa and storage moduli of 523.20 and 7012.25 Pa, respectively. Both hydrogels had a spontaneous and almost immediate swelling in aqueous media, and a high-water retention capacity (>80%), after 30 min incubation at 37 °C. Nevertheless, the Na5_1_*^4^ hydrogels had higher fatigue resistance and slightly higher-water retention capacity. These hydrogels were loaded with caffeine, ibuprofen, diclofenac, or salicylic acid, reaching entrapment efficiency values ranging between 13.11 ± 0.49% for caffeine, and 15.15 ± 1.54% for salicylic acid. Similar release profiles in PBS were observed for all tested APIs, comprising an initial fast release followed by a steady slower release. In vitro permeation experiments through Strat-M membranes using Franz diffusion cells showed considerably higher permeation fluxes for caffeine (33.09 µg/cm^2^/h) and salicylic acid (19.53 µg/cm^2^/h), compared to ibuprofen sodium and diclofenac sodium (4.26 and 0.44 µg/cm^2^/h, respectively). Analysis in normal human dermal fibroblasts revealed that CGC hydrogels have no major effects on the viability, migration ability, and morphology of the cells. Given their demonstrated features, CGC hydrogels are very promising structures, displaying tunable physical properties, which support their future development into novel transdermal drug delivery platforms.

## 1. Introduction

Hydrogels are hydrophilic structures with the ability to absorb large amounts of water inside their three-dimensional polymeric network [1,2,3]. Their structure is maintained by chemical or physical crosslinking of the polymer chains. Chemical crosslinking, present in the so-called chemical hydrogels, is characterized by the presence of covalent bonds and it involves the addition of crosslinking agents, which are often toxic thus affecting the hydrogels’ biocompatibility [4,5]. On the other hand, when polymer chains are physically cross-linked, non-covalent interactions are present and physical hydrogels are formed [2,6].

Polysaccharides emerged as very promising materials for the fabrication of hydrogels due to their nontoxicity, biocompatibility, biodegradability, and affordability [7,8]. These features allow their use in a wide range of applications in several areas, such as food [9], agriculture [10,11], and biomedical [12]. In particular, polysaccharide-based hydrogels have demonstrated great potential as delivery platforms for controlled drug delivery [13,14]. As drug delivery systems, polysaccharide-based hydrogels present improved functionalities such as stimuli-responsiveness, sustained drug release, target specificity, and therapeutic efficacy [15,16].

Chitin-glucan complex (CGC) is a water-insoluble and highly hydrophilic polysaccharide composed of chitin covalently linked to β-glucan chains [17,18]. As a major component of the cell wall of most yeasts and fungi, CGC provides stiffness and rigidity to the cell [19]. CGC merges the bioactive properties of β-glucan and chitin, being biodegradable, biocompatible, and exhibiting antibacterial, antioxidant, and anti-inflammatory properties [20,21,22]. CGC hydrogels can be fabricated by polymer coagulation from an alkali solution [23]. The procedure involves CGC dissolution in alkali solvent systems based on NaOH [24], to which freeze-thaw cycles are applied. CGC-based physical hydrogels spontaneously form during dialysis of the CGC alkali solution in water. This methodology was optimized in terms of freeze-thaw cycles and total freezing time, thus reducing the overall hydrogel preparation procedure time without impacting on their physical properties [24]. The resulting CGC-based hydrogels were evaluated as a drug delivery system, using caffeine as a model drug.

In this study, the procedure was further optimized by increasing the CGC concentration used for hydrogel fabrication, whose morphology, mechanical, and rheological properties were assessed. Furthermore, the hydrogels’ drug loading and release were studied, and permeation studies were performed to evaluate the potential of CGC hydrogels to be used as topical or transdermal delivery platforms.

## 2. Materials and Methods

### 2.1. Materials

CGC with a chitin content of 35.6% was extracted from the yeast *Komagataella pastoris* (DSM 70877) produced as described by Araújo et al. [25]. Caffeine (99%), diclofenac sodium salt (98%), ibuprofen sodium salt (98%), and salicylic acid (99%) were purchased from Alfa Aesar, Tokyo Chemical Industry Co, Sigma-Aldrich, and BDH, respectively.

### 2.2. Preparation of the CGC Hydrogels

The CGC hydrogels were prepared as described by Araújo et al. [24], with slight modifications. Briefly, the CGC powder was dispersed in a NaOH 5 mol/L solution, at two biopolymer concentrations (4 and 6 wt%), and the suspensions were kept at −20 °C. After that, the samples were thawed, extensively stirred (500 rpm, 1 h), at room temperature, and frozen again at −20 °C until achieving a total freezing time of 18 h. The undissolved fraction was removed from the suspensions by centrifugation (at 40,000 or 60,000× *g* for the 4 and the 6 wt% solutions, respectively), for 30 min, at 8 °C. The hydrogels were obtained by coagulation of the biopolymer during dialysis (12–14 kDa MWCO membranes, Spectra/Por, Spectrum Laboratories Inc., Piscataway, NJ, USA) of the alkali solutions, against deionized water, as described by Araújo et al. [23]. The hydrogels prepared with 4 and 6 wt% were labeled as Na5_1_*^4^ and Na5_1_*^6^ hydrogels, respectively.

### 2.3. CGC Hydrogels Characterization

#### 2.3.1. Chemical Characterization

The hydrogels’ water content was determined gravimetrically by freeze drying the hydrogel samples, following Equation (1):

(1) $$Water\;content=\frac{W_{wet}-W_{dry}}{W_{wet}}\times 100$$

where *W_dry_* (g) represents the dry mass of a pre-weighed hydrogel sample (*W_wet_*, g).

The chitin content (*Q*, %) was determined by elemental analysis (Flash EA 1112 Series CHNS analyzer, Thermo Scientific) based on the samples’ hydrogen content (*N*, %), using Equation (2):

(2) Q=14.199×N

#### 2.3.2. Morphology, Density and Porosity

The morphology and structure of the Na5_1_*^4^ and Na5_1_*^6^ hydrogels were analyzed by scanning electron microscopy (SEM) with a Tabletop Microscope TM3030 (Hitachi in High Technologies, Tokyo, Japan) equipped with a sample holder with refrigeration. The observations were performed at −4 °C, using magnifications of 500× and 1500×.

The hydrogel’s density (*ρ*, g/cm^3^) was assessed by gravimetry, following Equation (3):

(3) $$\rho =\frac{W_{dry}}{V_{dry}}$$

where *W_dry_* and *V_dry_* represent the weight (g) and volume (cm^3^) of the hydrogel, respectively.

The porosity of the hydrogels was determined by the solvent replacement method as described by Araújo et al. [24]. Briefly, pre-weighed freeze-dried hydrogel samples (*W*_0_, g) were immersed in absolute ethanol for 30 min, and after that time, excess ethanol was removed and the samples were weighed (*W*_30_, g). The porosity (%) was determined using Equation (4):

(4) $$Porosity=\frac{W_{30}-W_{0}}{\rho \;V_{T}}$$

where *ρ* is the density of ethanol (0.790 g/cm^3^) and *V_T_* (cm^3^) is the total volume of the hydrogel sample.

### 2.4. Compressive Mechanical Analysis

The compressive mechanical properties of the CGC hydrogels were assessed with a texture analyzer TMS-Pro (Food Technology Corporation, Slinfold, West Sussex, UK) equipped with a 50 N load cell. To perform uniaxial compressive tests, hydrogel cylindrical samples (13.7 mm diameter, 0.7–1.1 cm height) were compressed to a maximum strain of 80%, at a speed rate of 60 mm/min, using an aluminum plunger (60 mm diameter). The hardness, toughness, and compressive modulus were determined as described by Araújo et al. [24]. The hysteresis test was performed by compressing the cylindrical hydrogel samples in loading cycles to a maximum compression of 50% strain, with a speed rate of 60 mm/min and unloading at the same rate. The dissipation energy was calculated by measuring the area between the loading–unloading curves. To prevent water evaporation during the test, a thin layer of paraffin oil was applied to the hydrogel surface. All the experiments were performed at room temperature (20 ± 0.2 °C).

### 2.5. Rheological Properties

The rheological properties of the samples were analyzed using a controlled stress rheometer (HAAKE MARS III, Waltham, MA, USA Thermo Scientific), as described by Araújo et al. [24]. Hydrogel samples (~3 mm thickness) were equilibrated at 25 ± 0.03 °C, for 5 min, and their viscoelastic properties were determined by applying frequency sweeps at a constant tension within the linear viscoelastic region, for a frequency range from 0.01 to 1 Hz.

### 2.6. Swelling and Water Retention Behavior

The swelling properties of the CGC hydrogels were assessed gravimetrically, as described in Araújo et al. [24]. Briefly, pre-weighed (*W_dry_*, g) cylindrical freeze-dried samples were immersed in deionized water, phosphate buffer saline (PBS), or NaCl 0.9%, for 10 min, at 37 °C. After that period, samples were carefully removed from the solutions, the excess water was removed using filter paper, and weighed (*W_swollen_*, g). The swelling ratio (*g_water_*/*g_polymer_*) of the hydrogels was calculated as:

(5) $$Swelling\;ratio=\frac{W_{swollen}-W_{dry}}{W_{dry}}$$

The water retention ability of the hydrogels was evaluated by incubation of swollen samples, at 37 °C, for 30 min. The water retention (%) was determined by Equation (6):

(6) $$Water\;retention=\frac{W_{30}}{W_{swollen}}\times 100$$

where *W_swollen_* and *W*_30_ represent the weight (g) of the swollen hydrogels and of the hydrogel after 30 min of incubation, respectively.

### 2.7. Preparation of API-Loaded CGC Hydrogels

The Na5_1_*^4^ hydrogels were individually loaded with four different APIs, namely, caffeine, diclofenac, ibuprofen, and salicylic acid by diffusion method. Given the different water solubilities of the four APIs, solutions of 1.0 wt% caffeine, 0.1 wt% diclofenac, 1.0 wt% ibuprofen, and 0.1 wt% salicylic acid were prepared. For API loading, pre-weighed cylindrical freeze-dried hydrogel samples were immersed in the corresponding solution, for 24 h, at room temperature. After that period, API-loaded hydrogel samples were removed from the solutions, blotted with filter paper, and weighed (*W_L_*, mg). Drug loading (*D_L_*, mg_API_/cm^3^) and entrapment efficiency (*EE*, %) were calculated with Equations (7) and (8) [26,27] as follows:

(7) $$DL=\frac{(W_{L}-W_{dry})\times C_{API}}{V}$$

(8) $$EE=\frac{W_{loaded}}{W_{API}}\times 100$$

where *W_dry_* (g) represents the initial mass of dry hydrogel, *C_API_* (wt%) corresponds to the concentration of the API solution, *W_loaded_* is the API-loaded weight, and *W_API_* (g) represents the mass of API available.

### 2.8. In Vitro Drug Release Studies

Drug release studies were performed by immersion of cylindrical freeze-dried API-loaded hydrogels in 100 mL of PBS (pH 7.4), at 37 °C, for 4 h, under constant stirring (100 rpm). At predetermined time intervals, samples of 2 mL of the receptor medium were withdrawn and the same volume was replaced with a fresh and preheated medium. APIs concentrations in the withdrawn solution were determined by UV-Vis spectrophotometer (CamSpec M509T, Leeds, UK), at a wavelength of 273 nm for caffeine, 275 nm for diclofenac, 264 nm for ibuprofen, and 296 nm for salicylic acid. The APIs‘ cumulative release values were fitted to the Korsmeyer–Peppas model, according to Equation (9) [28]:

(9) $$\frac{M_{t}}{M_{\infty }}=kt^{n}$$

where *M_t_* and *M*_∞_ represent the amount of API (g) released at time *t* and infinite time, respectively; *k* is the kinetic constant characteristic of the drug–polymer interaction; and *n* is an empirical parameter for the release mechanism. According to this model, for cylinder samples, when *n* ≤ 0.45 the diffusion mechanism follows the Fickian diffusion (controlled diffusion), 0.45 < *n* < 0.89 is characteristic of a non-Fickian diffusion (anomalous transport), and *n* ≥ 0.89 represents a relaxation-controlled diffusion (controlled swelling) [28].

### 2.9. In Vitro Drug Permeation Studies

The drug permeation studies were performed using a Franz diffusion cell (PermeGear Inc., Hellertown, PA, USA) with a 3.14 cm^2^ diffusion area and a receptor volume of 10 mL. Strat-M membranes (Merck Millipore, Darmstadt, Germany) were used as skin-mimic artificial membranes. Prior to the diffusion experiments, the membranes were soaked in PBS (pH 7.4), at room temperature, for 12 h. After that period, the membranes were placed between the donor and the receptor compartments, and the latter was filled with PBS. The system was maintained under constant magnetic stirring (500 rpm), and the temperature was controlled at 37 °C by a circulating water bath.

In each experiment, a cylindrical API-loaded hydrogel sample (1.7 cm diameter; 0.3–0.5 cm thickness) was applied to the membrane. To minimize evaporation, the donor compartment and sampling port were occluded with parafilm. Periodically, a 400 µL sample was collected from the receptor compartment, and the same volume was replaced with a fresh and preheated receptor solution. Ibuprofen, caffeine, and diclofenac concentrations in the withdrawn solution were determined by high-performance liquid chromatography (HPLC) (Dionex Summit, Sunnyvale, CA, USA) using an Eclipse C18 column 4.6 × 250 mm (Agilent, Santa Clara, CA, USA) equipped with an amperometric detector. The analysis was performed at 25 °C, with acetonitrile-dipotassium hydrogen phosphate (65:35 *v*/*v*) as eluent, at a flow rate of 0.7 mL/min. The salicylic acid concentration in the withdrawn solution was determined by HPLC, using a Luna C18 column 4.6 × 250 mm (Phenomenex, Torrance, CA, USA). The mobile phase was a mixture of acetonitrile (ACN) and 0.1% of trifluoroacetic acid (TFA) used with the concentration of ACN varying from 12.5 to 100 to 12.5% in 0.1% TFA with the flow rate of 0.5 mL/min. The analysis was performed at 25 °C.

### 2.10. CGC Hydrogels Effect on Fibroblasts

For the wound scratch assay, normal human dermal fibroblasts, acquired from ATCC (PCS-201-010, Manassas, VA, USA), were seeded on a 35 cm^2^ tissue plate at a cell density of 4 × 10^5^ cells/plate, in Dulbecco’s modified Eagle medium (DMEM, Thermo Fisher Scientific, Waltham, MA, USA) supplemented with 10% (*v*/*v*) fetal bovine serum (Thermo Fisher Scientific) and a mixture of 100 U/mL penicillin and 100 μg/mL streptomycin, and incubated at 37 °C, 5% (*v*/*v*), and 99% (*v*/*v*) relative humidity, until confluence was reached. A scratch on the confluent monolayer was performed using a sterile micropipette tip, the medium was replaced by fresh medium and a sterilized CGC hydrogel freeze-dried sample with 5 mm diameter was placed over the scratch. For control purposes, a scratch was also done on another tissue plate that was not submitted to the presence of the CGC hydrogel. Regions of the scratch were imaged at 0 h and after 12 h with a Cytosmart Lux2 (Cytosmart technologies, Eindhoven, The Netherlands) and the size of the wound scratch was measured using ImageJ software.

The morphology of fibroblast cells after exposure to the hydrogel sample was evaluated by confocal microscopy. After measuring the scratch, the cells were fixed with 4% (*w*/*v*) formaldehyde, washed 3 times with PBS, and then permeabilized for 5 min with 0.1% (*v*/*v*) Triton X-100. Afterwards, the cells were incubated with 1% (*w*/*v*) bovine serum albumin, actin was stained with AlexaFluor 488 Phalloidin (Thermo Fisher Scientific) according to the manufacturer’s procedure recommendations, and nuclei were stained with 7.5 μg/mL Hoechst 33258 (Thermo Fisher Scientific). Cells were visualized in a Zeiss LSM 710 confocal microscope, and 5 different images of cells exposed to CGC hydrogel were acquired using microscope software (Zen black edition, 2011).

Fibroblasts’ viability was evaluated by preparing 4 wells in a 24-well plate, 2 with 7500 cells/well and 2 without cells. After 24 h for cell adherence, the CGC hydrogel sample (5 mm in diameter) was added to a well with cells and to a well without cells. After 24 h, the medium was replaced by fresh medium supplemented with 3-(4,5-dimethylthiazol-2-yl)-5-(3-carboxymethoxyphenyl)-2-(4-sulfophenyl)-2H tetrazolium, inner salt (MTS, Promega, Madison, WI, USA), and the absorbance was measured at 490 nm, according to manufacturer’s instructions. The percentage of fibroblasts’ viability exposed to CGC hydrogel was measured using Equation (10) as follows:

(10) $$\%\;viability=\frac{Abs_{490}\;fibroblasts\;with\;CGC\;hydrogel-Abs_{490}\;medium\;with\;CGC\;hydrogel}{Abs_{490}\;fibroblasts-Abs_{490}\;medium}\times 100$$

### 2.11. Statistical Analysis

The experimental data from all the studies were expressed as mean ± standard deviation (SD). Error bars represent the standard deviation (*n* ≥ 3).

## 3. Results

### 3.1. Hydrogels Fabrication

**Figure 1:**
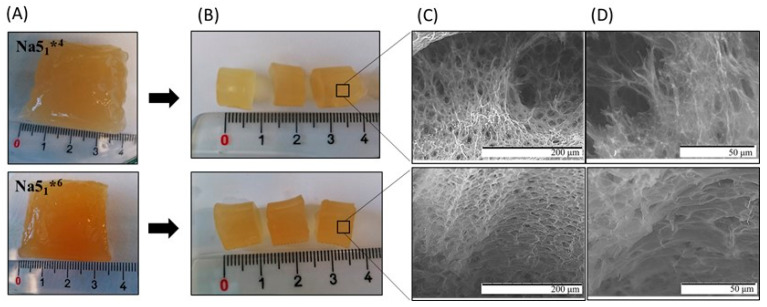
Na5_1_*^4^ and Na5_1_*^6^ hydrogels: (**A**) immediately after dialysis, (**B**) after being cut with cylindrical mold, and (**C**,**D**) SEM images of the hydrogels. Hydrogel regions observed under 1500× magnification (**D**) were expanded from images presented under 500× magnification (**C**).

The CGC hydrogels, named Na5_1_*^4^ and Na5_1_*^6^ (Figure 1A), prepared from 4 and 6 wt% CGC suspensions, respectively, had polymer contents of 2.40 ± 0.15 wt% and 3.09 ± 0.22 wt% (Table 1), respectively, which shows that the CGC content of the suspensions was not completely solubilized in the NaOH solution. In fact, around 60% of the polymer in the 4 wt% suspension was dissolved in NaOH, while it decreased to 51.5% for the 6 wt% suspension. Compared to previous work, where hydrogels with a polymer content of 1.66 ± 0.11 wt% were obtained from a 2 wt% CGC suspension in NaOH 5 M (named Na5_1_* hydrogels) [24], corresponding to a dissolution of 83% of the biopolymer, it is clear that increasing the initial CGC content in the alkali suspension results in lower solubilization of the biopolymer. Nevertheless, the obtained hydrogels had higher polymer content (Table 1). As shown in Table 1, all CGC hydrogels presented characteristic high water contents, above 96% (Table 1), and had similar chitin contents (20.9 ± 0.78 and 21.4 ± 0.71%, for the Na5_1_*^4^ and Na5_1_*^6^ hydrogels, respectively).

The Na5_1_*^6^ hydrogels revealed a slightly lower porosity (72.0 ± 0.43%) than the Na5_1_*^4^ hydrogels (79.4 ± 0.60%) (Table 1), but considerably higher than the value reported for the Na5_1_* hydrogels (53.8 ± 10.3%) [24]. The higher CGC concentration of the Na5_1_*^6^ hydrogels resulted in structures with a higher density (0.052 ± 0.010 g/cm^3^) compared to the values found for the Na5_1_*^4^ (0.037 ± 0.005 g/cm^3^) and the Na5_1_* (0.019 ± 0.001 wt%) hydrogels (Table 1). The porosity and density of hydrogels are essential parameters determined by their microstructure that significantly influence their physicochemical properties and their loading and release ability [29].

### 3.2. Morphological Characterization

Figure 1 shows the macroscopic appearance of the CGC hydrogels immediately after the removal from the dialysis membrane (Figure 1A) and after being cut into small samples (13.7 mm diameter, 0.7–1.1 cm height) (Figure 1B), by the use of a cylindrical mold to obtain samples with similar dimensions. Macroscopically, no significant differences were observed between the Na5_1_*^4^ and Na5_1_*^6^ hydrogels. The fresh hydrogels were translucid and exhibited a yellowish coloration (Figure 1A,B). However, a more intense coloration was observed for the Na5_1_*^6^ hydrogels, which can be related to their higher polymer content.

SEM analysis (Figure 1C,D) revealed that for both hydrogels the polymeric chains formed a heterogeneous and compact three-dimensional network, which is concomitant with the structures previously reported for CGC hydrogels [23,24]. However, the Na5_1_*^4^ hydrogels presented more irregular and open regions, while a regular and consistent microstructure was observed for the Na5_1_*^6^ hydrogels (Figure 1C). Upon magnification, smaller pores were observed for the Na5_1_*^6^ hydrogels (Figure 1D) which explains the denser and tighter microstructure of these structures. Similar observations were reported for chitosan [30] and cellulose hydrogels [31] upon increasing polymer concentration. The denser inner structure of the Na5_1_*^6^ sample can be explained by the higher physical crosslinking and chain entangling of CGC molecules displayed by those hydrogels. The observed lower pore size and denser structure of the Na5_1_*^6^ hydrogels are related to their higher polymer content [32]. These observations are in line with the increased density and lower porosity values obtained for the Na5_1_*^6^ hydrogels (Table 1).

### 3.3. Mechanical Properties

**Figure 2:**
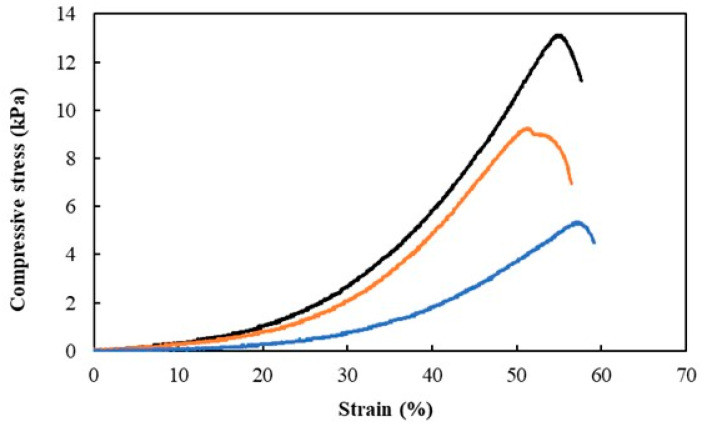
Compression stress–strain curves of Na5_1_* (blue line), Na5_1_*^4^ (orange line), and Na5_1_*^6^ (black line) hydrogels.

**Figure 3:**
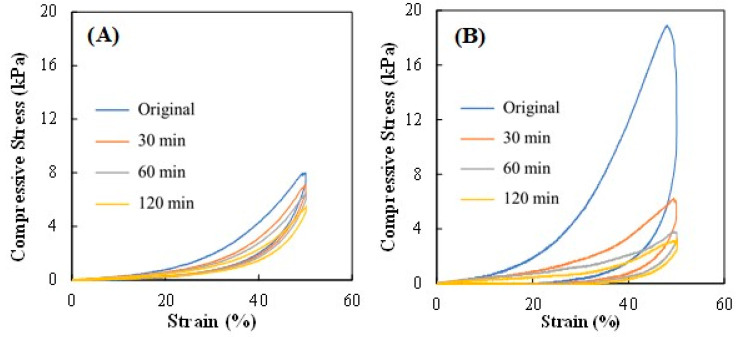
Loading–unloading curves of (**A**) Na5_1_*^4^ and (**B**) Na5_1_*^6^ hydrogels, under 50% strain.

The mechanical properties were evaluated by performing two types of experiments, namely, the application of a single 80% strain compression (Figure 2) to determine the hydrogels’ hardness, compressive modulus, and toughness (Table 1), and the application of cyclic compressive tests (Figure 3A,B) to evaluate the dissipation energy (Table 1). As shown in Table 1, the highest hardness (13.16 ± 0.15 kPa), compressive modulus (47.30 ± 2.04 kPa), and toughness values (2.14 ± 0.12 kJ/m^3^) were observed for the Na5_1_*^6^ hydrogels. This result is probably related to the higher polymer content of those hydrogels (3.09 ± 0.22 wt%) compared to the Na5_1_*^4^ and Na5_1_* hydrogels (2.40 ± 0.15 and 1.66 ± 0.11 wt%, respectively) (Table 1). Liu et al. [33] reported higher hardness (2.0 kPa) for carboxymethyl chitin 2.0 wt% hydrogels, compared to 0.5 kPa for hydrogels with 1.0 wt% polymer content. Similar behavior was also demonstrated for gelatin methacrylate hydrogels, where the compressive modulus was significantly improved when the polymer concentration was increased from 5 to 10% [32]. In general, these results can be explained by the increase in multiple physical interaction sites and entrapped entanglements resulting from increased polymer concentration [34].

Despite this, all samples displayed similar compressive stress–strain curves (Figure 2), characteristic of non-linear and viscoelastic solids [35], for which the rupture strain occurred between 50 and 60%. These results suggest that increasing the polymer concentration in the CGC hydrogels has no significant impact on their capacity to withstand deformation.

The cyclic compressive tests, in which the wet hydrogel samples were subjected to loading cycles at increasing periods of time (30, 60, and 120 min) at maximum compression of 50% strain, revealed that the Na5_1_*^6^ hydrogels presented higher dissipation energy values than the Na5_1_*^4^ hydrogels (Table 1), for all tested loading times, despite decreasing with increasing loading cycles. In fact, the original Na5_1_*^6^ hydrogel sample presented a dissipation energy of 2.38 ± 0.56 kJ/m^3^, while after 120 min the value decreased to 0.25 ± 0.06 kJ/m^3^. The Na5_1_*^4^ hydrogels, on the other hand, although having a lower original dissipation energy (0.38 ± 0.09 kJ/m^3^), were less impacted by the consecutive loading cycles, displaying a value of 0.19 ± 0.001 kJ/m^3^ after 120 min (Table 1). These results indicate that increasing the polymer concentration led to a more efficient energy dissipation of the Na5_1_*^6^ hydrogels, but the efficiency decreased by applying more cycles.

The compression loading–unloading curves are shown in Figure 3A,B. It can be observed that the loading curves differed from the unloading curves for all the tested periods of time. Moreover, the hysteresis loops or the area of loading and unloading closed curves, which reflected the dissipated energy, decreased as the number of cycles increased for all hydrogel samples. Nonetheless, the Na5_1_*^4^ hydrogels had a better recovery behavior as shown by the compressive stress values that decreased from 7.99 kPa for the first cycle to 5.56 kPa for the fourth cycle (Figure 3A), compared to the Na5_1_*^6^ hydrogels for which a more significant decrease was observed (from 18.91 to 6.25 kPa, for the first and second cycles, respectively) (Figure 3B). A similar behavior was reported by Shen et al. [36] for chitosan-gelatin hydrogels, for which a decrease in the hysteresis loop area was observed, followed by a plateau value, as compressive cycles increased. The authors attributed this behavior to the remarkable fatigue resistance of the hydrogels.

For the Na5_1_*^6^ hydrogels, there was a drastic decrease in the dissipated energy and compressive stress. As shown in Figure 3B, a large hysteresis was obtained for the first cycle, corresponding to a compressive stress of 18.91 kPa, which decreased to 6.25, 3.83, and 3.36 kPa for the subsequent cycles. This significant concomitant reduction in dissipated energy with a decrease in strength might be attributed to a low recovery ability due to the internal fracture of the hydrogel structure during the first loading–unloading cycle [37]. These results show that, although the Na5_1_*^6^ hydrogels were tougher, probably due to their higher polymer content, the Na5_1_*^4^ had higher fatigue resistance.

### 3.4. Rheological Properties

**Figure 4:**
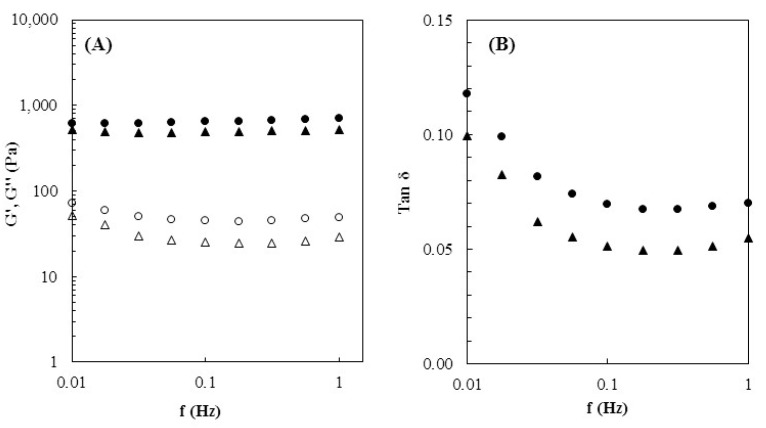
Rheological properties of the Na51^*4^ (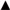) and Na51^*6^ (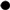) hydrogels at 25 °C: (**A**) Mechanical spectrum storage (G′, solid symbols) and loss moduli (G″, open symbols), (**B**) loss tangent (tan δ).

As shown in Figure 4A, both hydrogels exhibited predominantly elastic characteristics, with the elastic (storage) modulus G′ exceeding in one order of magnitude their corresponding viscous (loss) modulus G″ over the whole range of frequencies. Both G′ and G″ were mainly frequency-independent, suggesting the formation of a stable structure [38]. Similar elastic behavior was previously reported for Na-based CGC hydrogels [23,24].

The Na5_1_*^6^ hydrogels exhibited higher values of both G′ and G″ across the entire range of frequencies (Figure 4A), which can be related to their higher polymer content. For a frequency of 1 Hz, the Na5_1_*^6^ hydrogels presented a G′ of 702.25 ± 28.65 Pa, while lower values were reached for the Na5_1_*^4^ hydrogels (523.20 ± 21.08 Pa), as well as for the Na5_1_* hydrogels (149.9 ± 9.8 Pa) [24]. This behavior can be explained by the denser cross-linked structure the Na5_1_*^6^ hydrogels formed (Figure 1C) due to the higher polymer concentration and has been reported for other CGC-based hydrogels. An example are the hydrogels prepared with *Ganoderma lucidium* CGC dissolved in ionic liquids [39], for which the authors reported a gel strength increase (from ~3500 to ~7000 Pa, at 1 Hz) by increasing the polymer concentration from 4 to 6 wt%.

The viscoelastic behavior of CGC hydrogels was also determined by the value of the loss tangent of delta (tan δ), which represents the ratio of energy lost to energy stored during deformation. As shown in Figure 4B, both hydrogels exhibited tan δ values below 1, which confirms the elastic characteristics of CGC hydrogels [40]. Moreover, it can be observed that by increasing the frequency a decrease in the tan δ values was achieved, and for both hydrogels, a plateau was reached. This behavior is associated with the mostly frequency-independent performance of both moduli (Figure 4A). Similar behavior was reported for chitosan-based hydrogels [41]; however, those structures were weaker than CGC hydrogels since higher tan δ values (above 0.15) were obtained. Over the whole range of frequencies, the Na5_1_*^4^ hydrogels presented lower tan δ values (0.049–0.100) compared to Na5_1_*^6^ hydrogels (0.067–0.118). The results indicate that during deformation, Na5_1_*^4^ hydrogels have more elastic characteristics, which give them a higher ability to store energy than to dissipate it. These findings are in accordance with the mechanical properties and revealed that increasing the polymer content led to the fabrication of more rigid hydrogels.

### 3.5. Swelling Ratio and Water Retention Behavior

**Figure 5:**
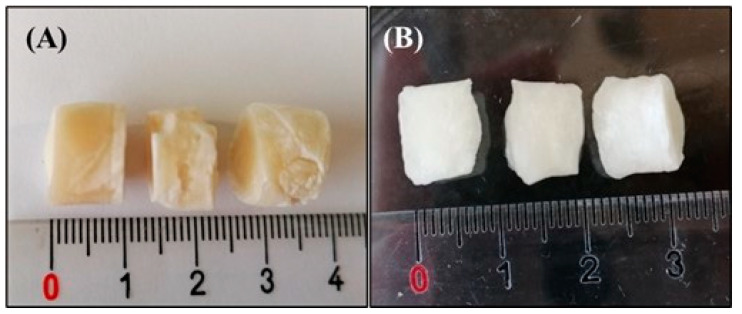
Macroscopic appearance of (**A**) freeze-dried and (**B**) swollen CGC hydrogel samples.

The swelling ability of CGC hydrogels was assessed by immersing freeze-dried hydrogel samples (Figure 5A) in three different media (deionized water, PBS, and NaCl 0.9%) at 37 °C, for 10 min (Figure 5B). Macroscopically, after freeze-drying, the structures’ dimensions were retained (Figure 5A), showing that the polymer network was not significantly affected by water removal. Moreover, the structures presented a lighter yellowish coloration and were rigid. Upon rehydration, in spite of the similar dimensions, hydrogels exhibited a whitish color and became opaque (Figure 5B).

**Figure 6:**
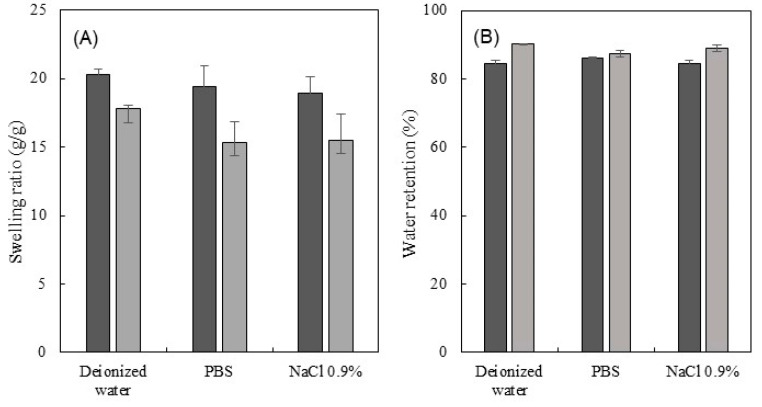
Swelling ratio (**A**) after 10 min and water retention capacity (**B**) after 30 min incubation of the Na5_1_*^4^ (dark grey) and the Na5_1_*^6^ (light grey) hydrogels, in deionized water, PBS, and NaCl 0.9%, at 37 °C.

As shown in Figure 6A, upon immersion in the aqueous media for 10 min, the Na5_1_*^4^ hydrogels presented a high swelling ratio, achieving values of 20.3 ± 0.4, 19.4 ± 1.5, and 19.0 ± 1.1 g/g for deionized water, PBS, and NaCl 0.9%, respectively. Due to their lower polymer content, these structures presented a decreased crosslink density and, consequently, higher pore sizes for water absorption, as demonstrated by the SEM images (Figure 1C,D). In contrast, for a similar time period, lower swelling ratio values (17.8 ± 0.3, 15.3 ± 1.5, and 15.5 ± 1.9 g/g, respectively) were obtained for the Na5_1_*^6^ hydrogels. In general, the obtained values for Na5_1_*^4^ and Na5_1_*^6^ hydrogels were lower than the ones reported for Na5_1_* hydrogels (28.6 ± 1.3, 21.0 ± 0.5, and 19.2 ± 0.5 g/g, respectively), which is in line with the lower polymer content of the latter (Table 1). Analogous performance was reported by Liu et al. [39] for the *Ganoderma lucidium* CGC hydrogels, whose swelling ratio also decreased (from 1181.0 to 1891.0%) as the polymer concentration increased from 2 to 7 wt%. However, in deionized water, the hydrogels prepared with 4 and 6 wt% of polymer exhibited lower swelling ratio values (~1400%, corresponding to 14 g/g) when compared to Na-based CGC hydrogels.

For all the tested media, both hydrogels exhibited exceptional water absorption ability (19.0–20.3 g/g and 15.3–17.8 g/g, for the Na5_1_*^4^ and the Na5_1_*^6^ hydrogels, respectively), due to the presence of hydroxy and amino groups in the CGC macromolecules that provided it high hydrophilicity and surface polarity [19]. Moreover, it can be observed that for both hydrogels the swelling ratio was higher in deionized water than in either PBS or NaCl 0.9% (Figure 6A). This behavior was also reported for Na5_1_* hydrogels and it can be related to the difference in osmotic pressure between the hydrogel’s structure and the saline solutions [24]. Furthermore, the neutral pH of the PBS solution (7.4) might induce an increase in the swelling ratio of hydrogels due to the deprotonation of -NH_3_ and the intra-chain hydrogen bonds in the hydrogel matrix [42]. Nonetheless, the slight difference between the swelling ratio in PBS and NaCl 0.9% suggested the absence of a pH-sensitive swelling behavior. Additionally, for both hydrogels, the swelling equilibrium was reached almost spontaneously in all the tested media, which demonstrates the sponge-like behavior of these structures (Figure 5).

For the assessment of the water retention ability, swollen samples of the three different media were incubated at 37 °C, for 30 min. At specific times, the water loss was evaluated by weighing the samples. Figure 6B shows the water retention ability of the Na5_1_*^4^ and Na5_1_*^6^ hydrogels. As demonstrated, after 30 min at 37 °C, both hydrogels were able to retain above 80% of their water content. Despite their lower swelling ratio, the Na5_1_*^6^ hydrogels had a higher water retention capacity (87.3–90.2%) than the Na5_1_*^4^ hydrogels (84.6–86.2%), which may be due to their more compact network structure. Nevertheless, for the tested period, no significant differences were observed between the hydrogels’ water retention ability in the different media.

The swelling behavior defines the water absorption capacity of hydrogels, and it has a direct impact on their ability to load and deliver drugs [43]. In fact, a conventional method for loading hydrogel structures is performed by soaking a preformed hydrogel in a drug solution and allowing it to swell to equilibrium, avoiding adverse effects on drug properties caused by polymerization [44]. The subsequent drug release includes the simultaneous absorption of water into the hydrogel matrix and the desorption of drugs via diffusion. Several studies demonstrated that higher swelling ability leads to increased drug loading and drug release rates. For example, Suhail et al. [45] reported a decreased drug loading capacity of chondroitin sulfate-based hydrogels that presented lower swelling when compared to the ones with higher swelling.

Given their interesting mechanical and rheological properties, particularly their higher fatigue resistance, as well as their higher swelling ability, the Na5_1_*^4^ hydrogels were selected for the subsequent tests, namely, APIs loading and in vitro release, and permeation tests.

### 3.6. Hydrogels Loading and Release Ability

#### 3.6.1. Loading of API

The drug loading capacity of the Na5_1_*^4^ was evaluated by soaking cylindrical freeze-dried hydrogel samples in four individual APIs solutions, namely, caffeine, salicylic acid, diclofenac sodium, and ibuprofen sodium, at room temperature, for 24 h. Table 2 shows that the higher concentration of caffeine and ibuprofen sodium loading solutions (1.0 wt%) led to a higher amount of those APIs within the hydrogel’s matrix (11.98 and 11.25 mg/cm^3^, respectively). On the other hand, due to the low water solubility of diclofenac sodium and salicylic acid, lower concentrations of those APIs (0.1 wt%) were used and, consequently lower drug loading capacity values were obtained (1.30 and 1.49 mg/cm^3^, respectively). In fact, the drug concentration of the loading solution has a direct effect on the hydrogels’ drug loading since the increase in the concentration of the drug leads to a higher amount of drug available to be loaded into the hydrogels’ structures. A similar effect has been reported for several drug delivery systems including calcium pectinate beads [46] and chitosan-silk fibroin films [47]. Indeed, the latter structures achieved an increase in diclofenac content from 1.075 to 4.564 µg/mm^3^ using drug loading solutions of 62.5 and 250 µg/mL, respectively.

Additionally, the loading method also significantly affects the drug loading content. For example, Sriamornsak et al. [46] reported a decrease in the drug content on calcium pectinate beads when the drug loading process was based on gel swelling (0.97–4.37 mg/g), compared to mixing (27.97–29.15 mg/g). Despite the higher values of drug content achieved, the processes used for the formation of CGC hydrogels preclude the use of mixing methods.

Moreover, it was noticed that the entrapment efficiency was also affected by the physicochemical characteristics of the different tested APIs. As shown in Table 2, caffeine reached the lowest value of EE% (13.11 ± 0.49%), which can be explained by the API ionization and the lower acetylation degree of the N-acetyl-glucosamine monomers of CGC [24]. On the contrary, the highest EE% values were obtained for salicylic acid, reaching values of 15.15 ± 1.54%, which might be justified by the low molecular weight (138.12 Da) of the API’s molecules. In fact, with the loading swelling method, the drug’s molecular weight has a significant impact on the drug loading process. For example, Caliceti et al. [48] reported a linear correlation between drug molecular weight and loading yield where a decrease in loaded drug amount was obtained as the drug molecular weight increased.

This behavior was also observed by Sethi et al. [49] who described a similar soaking method of loading xanthan gum-starch hydrogels with paracetamol and aspirin, and higher EE% values were achieved (70.12 and 62.14%, respectively). Despite the low solubility of aspirin, its higher molecular weight might have a negative impact on the loading process. Wong et al. [50] reported a higher loading percentage of ibuprofen (59.08 ± 3.97%) compared to diclofenac (20.68 ± 0.47%) in poly(ethylene oxide) hydrogels using loading solutions with concentrations of 8.01 and 1.98% *w*/*v*, respectively. In this case, the use of higher concentrations of loading solutions may contribute to the increased values achieved compared to CGC-based hydrogels.

#### 3.6.2. In Vitro Release Studies

**Figure 7:**
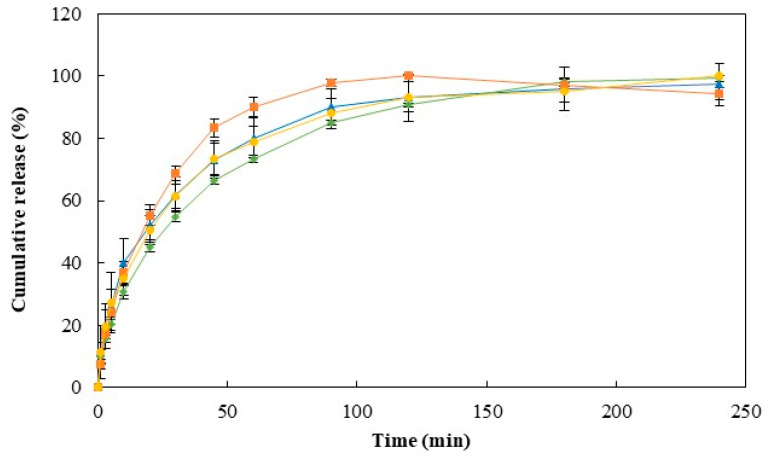
Cumulative release profile of caffeine (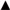, blue line), diclofenac sodium (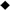, green line), salicylic acid (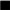, orange line), and ibuprofen sodium (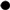, yellow line) of Na5_1_^*4^ hydrogels in PBS, at 37 ° C.

The release studies were conducted by placing the API-loaded Na5_1_*^4^ hydrogels in PBS solution, at 37 °C. The cumulative release profiles of the four APIs are shown in Figure 7. It can be observed that hydrogels presented a release profile predominantly controlled by diffusion, characterized by an initial burst release [51]. For all the APIs tested, within 3 h of the experiment, the amount of API loaded in the structure of the hydrogel was totally released. An analogous drug release profile has been previously reported for caffeine-loaded CGC hydrogels [24]. However, due to their higher polymer content and consequent lower pore size, slightly lower release rates were displayed by the Na5_1_*^4^ hydrogels compared to the Na5_1_* hydrogels. In particular, the latter achieved a caffeine release of around 50% after 10 min in the same release media, while values of 40% were reached for Na5_1_*^4^ hydrogels. A similar behavior was described for the polyacrylamide hydrogels that presented a 30% decrease in drug delivery rate when polymer concentration was increased from 2.5 to 10% [52].

Pore dimensions determine the diffusion of the drug through the hydrogel since it controls the steric interactions between the drug and the polymer network [51]. Moreover, when the drug molecules are smaller than the hydrogels’ pores, a fast diffusion occurs, and a short release duration is obtained. Figure 7 shows that as the molecular weight of the API increased, a slight decrease in the drug release was observed. Indeed, salicylic acid, which was the API with the lower molecular weight, presented the highest release rate. On the other hand, the slowest release rate was obtained for diclofenac, which has a higher molecular weight. As shown in Figure 7, within 1 h, a salicylic acid release percentage of 90.11 ± 3.11% was achieved, whereas a lower value (73.38 ± 1.02%) was reached for diclofenac sodium. For the same period and analogous released media, slower released rates were reported by Gull et al. [53] for the chitosan-based hydrogels, whose diclofenac release achieved values between 30 and 50%. However, a similar release percentage (>90%) was obtained after 130 min of the experiment. This difference might be attributed to the higher swelling ability of CGC-based hydrogels, at pH 7, which allows an increase in the drug release rate from the structure [45].

The drug release kinetics was evaluated by fitting the first initial 60% of the API released to the Korsmeyer–Peppas model [28]. Table 2 demonstrates that all the obtained release data fitted in the model since the regression coefficients (R^2^) were around 0.99. Moreover, according to the kinetic parameters obtained, all the API releases followed a non-Fickian diffusion or anomalous transport, since the *n* values are between 0.45 and 0.89. These results indicate that drug diffusion and polymer chain relaxation contribute to the overall release rate [50].

It can be observed that the release profile of caffeine from Na5_1_*^4^ hydrogels presented an *n* value of 0.523, which is a higher value when compared to the one reported for Na5_1_* hydrogels (0.42) [24]. The prior structures presented a slow release of the API and the mechanism deviated from the controlled diffusion, which justifies the higher *n* values obtained. A non-Fickian transport of caffeine and diclofenac molecules from bacterial nanocellulose membranes was also reported by Silva et al. [27]. In this case, *n* values obtained for those APIs were 0.52 and 0.55, respectively, which are similar to the ones obtained for Na5_1_*^4^ hydrogels (Table 2).

### 3.7. In Vitro Permeation Studies

**Figure 8:**
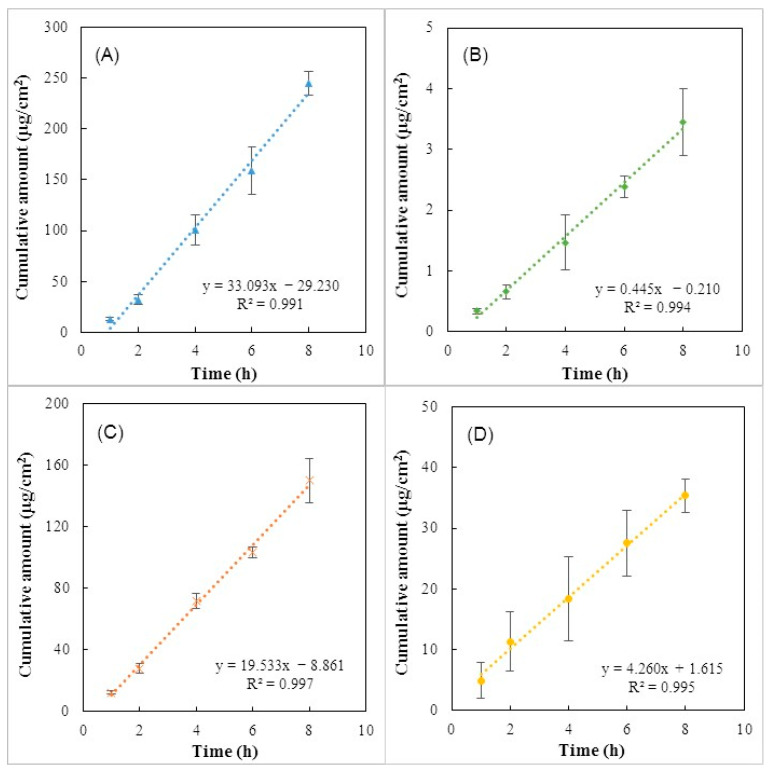
Cumulative amount profiles of (**A**) caffeine, (**B**) diclofenac sodium, (**C**) salicylic acid, and (**D**) ibuprofen sodium from Na5_1_*^4^ hydrogels.

Figure 8 shows the receptor cumulative amount profiles of the different tested APIs across mimic-skin artificial membranes (Strat-M), which are an effective alternative to human or animal skin to predict in vivo drug transdermal diffusion [54,55]. It can be noticed that all APIs were able to permeate the membrane, and this fact can be explained by their low molecular weights (138.12–318.13 Da). Indeed, for a drug to be passively delivered across the skin, the molecular weight should be below 500 Da [56,57]. As shown in Figure 8, the maximum cumulative amount of 245.02 ± 11.17 µg/cm^2^ was achieved for caffeine (Figure 8A), while the lowest value was reached for diclofenac (3.45 ± 0.55 µg/cm^2^) (Figure 8B). Figure 8C,D, shows that salicylic acid also achieved a high cumulative amount value (150.05 ± 14.24 µg/cm^2^) and a low cumulative amount was obtained for ibuprofen (35.37 ± 2.77 µg/cm^2^). This difference can be explained by the ionization properties of each API and the interaction between the API and the polymeric chains. Ionized drugs are poor candidates for transdermal delivery due to the lipophilic nature of the *stratum corneum* [56,58]. Among the APIs used, caffeine represents the only non-ionic molecule, which might have improved its permeation through the membrane, resulting in the highest cumulative amount achieved. For example, Uchida et al. [59] reported a permeability coefficient of unionized lidocaine 43-fold higher than the value obtained for the ionized form of the molecule. Besides their ionization, salicylic acid, ibuprofen, and diclofenac are negatively charged molecules, which make them suitable for interactions with polymer chains and, consequently, decrease their permeation.

One important permeation parameter is the steady-state flux (Jss), corresponding to the amount of permeate crossing the membrane at a constant rate that can be obtained from the slope of the linear region of the cumulative amount over time [60].

As shown in Figure 8, caffeine presented the highest permeation with a Jss value of 33.09 µg/cm^2^/h followed by salicylic acid and ibuprofen, which were permeated with a flux of 19.53 and 4.26 µg/cm^2^/h, respectively. On the other hand, the lowest permeation was achieved by diclofenac sodium with a Jss value of 0.44 µg/cm^2^/h. As explained above, these results can be attributed to the physicochemical nature of the API, namely, the ionization, the molecular mass, and the partition coefficient. The partition coefficient is a key parameter for skin permeation since it determines which pathway a drug molecule would take after passing through the *stratum corneum* [56]. Moreover, the partitioning of a drug between the lipophilic *stratum corneum* and the hydrophilic living cells underlying the epidermis can be well represented by the *n*-octanol-water partition coefficient (logP) [56]. Among the ionized APIs used, salicylic acid exhibited a logP value of 2.26, which is within the ideal range (1 < logP < 3) established for transdermal permeation [57]. This characteristic might explain the high cumulative amount achieved and the increased value of permeation flux (Figure 8C). Additionally, the low molecular weight of salicylic acid also promotes its permeation. Uchida et al. [61] demonstrated a correlation between the permeated amount and molecular weight, where the permeated amount decreased with an increase in the molecule mass. This fact might explain the low cumulative amounts achieved for ibuprofen and diclofenac, which are molecules with higher molecular weight (229.27 and 318.13 Da, respectively) when compared to the salicylic acid molecule (138.12 Da). In comparison with diclofenac, ibuprofen achieved higher permeation flux, and this might be attributed to the higher drug concentration used in loading solutions (Figure 7A). Pradal et al. [62] also reported that increasing the drug content in the formulations, led to an enhancement of the permeation. These results demonstrated that CGC hydrogels can be used as a platform to deliver drugs with different physicochemical characteristics.

### 3.8. Effect of CGC Hydrogels in Fibroblasts

**Figure 9:**
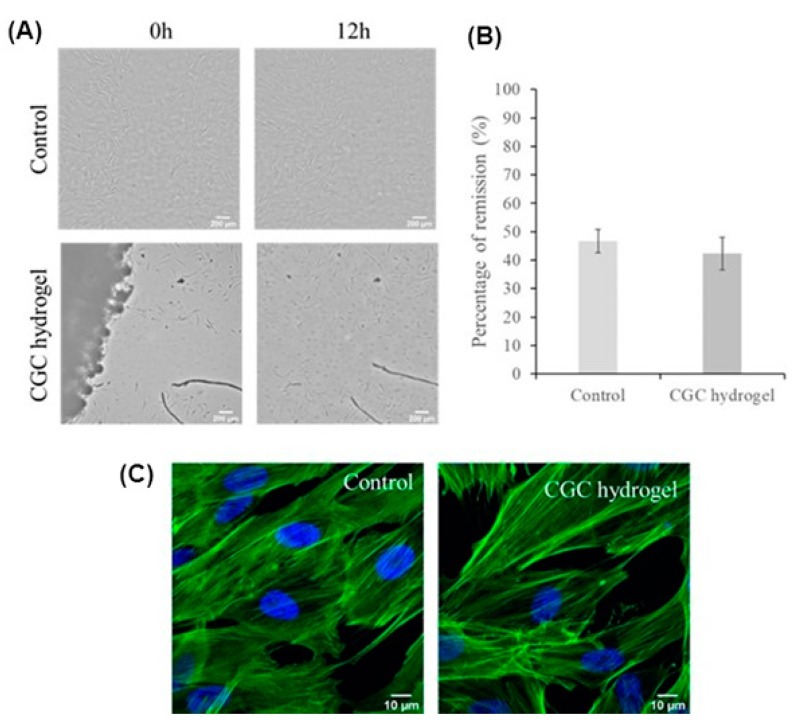
Representative images of the wound healing assay (**A**) at 0 h and 12 h after performing a scratch in a fibroblasts monolayer in the presence of a CGC hydrogel (CGC hydrogel) or absence (control); percentage of remission (**B**) after 12 h exposure to CGC hydrogel, or untreated (control). Bars represent the mean ± SEM of two independent experiments. Aspect of fibroblasts (**C**) in the absence (control) or presence (CGC hydrogel) of the CGC hydrogel. Cells were exposed for 12 h to the freeze-dried CGC hydrogel sample, fixed with 4% (*w*/*v*) formaldehyde, and then actin filaments were stained with phalloidin conjugated with AlexaFluor 488 (green fluorescence) and nuclei stained with Hoechst 33258 (blue fluorescence). The presented images are representative of five images acquired with a Zeiss LSM710 confocal microscope.

To assess the influence of the CGC hydrogels in tissue regeneration, a wound scratch assay was performed with human normal primary dermal fibroblasts in the presence and absence of the hydrogel, according to previously described procedures [63]. No major differences were observed in the remission percentage when the cells were exposed or not exposed to the hydrogel samples (Figure 9A,B).

A deeper look into the cells’ appearance after actin and nuclei staining also revealed that no major differences were noticed in the morphology of the connective tissue cells after exposure to the CGC hydrogel (Figure 9C), suggesting that it has no significant impact on these dermal cells. In line with these results, an 88.8 ± 3.5% viability was observed for the fibroblasts exposed to the CGC hydrogel samples, compared to the unexposed cells.

## 4. Conclusions

This study demonstrated that increased CGC content led to the formation of denser hydrogels with improved mechanical and rheological properties. However, the more compact polymeric network of the Na5_1_*^6^ hydrogels was characterized by lower fatigue resistance and lower swelling ability compared to the Na5_1_*^4^ hydrogels. The latter revealed a high loading ability for several APIs, namely, caffeine, salicylic acid, diclofenac sodium, and ibuprofen sodium, displaying a release profile following a non-Fickian diffusion. Drug loading and release were dependent on the API’s molecular weight and ionic character. Analysis in normal human dermal fibroblasts revealed that CGC hydrogels have no major effects on the viability, migration ability, and morphology of cells, which is an indicator of their non-cytotoxicity. This study highlights the use of CGC-based hydrogels as drug delivery systems and their potential as promising structures to be used in biomedical applications.

## Figures and Tables

**Table 1:** Chemical characterization, compressive mechanical properties (under 80% strain), and dissipation energies (cycles under 50% strain) of Na5_1_*^4^ and Na5_1_*^6^ hydrogels.

Samples	Na5_1_*	Na5_1_*^4^	Na5_1_*^6^
CGC_initial_ (wt%)	2	4	6
CGC_hydrogel_ (wt%)	1.66 ± 0.11	2.40 ± 0.15	3.09 ± 0.22
Water content (wt%)	97.63 ± 0.12	97.60 ± 0.15	96.91 ± 0.22
Porosity (%)	53.8 ± 10.3	79.4 ± 0.60	72.0 ± 0.43
Density (g/cm^3^)	0.019 ± 0.001	0.037 ± 0.005	0.052 ± 0.010
Hardness (kPa)	5.04 ± 0.14	10.08 ± 0.89	13.16 ± 0.15
Compressive modulus (kPa)	23.00 ± 0.89	39.36 ± 0.36	47.30 ± 2.04
Toughness (kJ/m^3^)	0.78 ± 0.015	1.61 ± 0.33	2.14 ± 0.12
Dissipation energy (kJ/m^3^)			
0 min	-	0.38 ± 0.09	2.38 ± 0.56
30 min	-	0.26 ± 0.02	0.70 ± 0.02
60 min	-	0.24 ±0.01	0.40 ± 0.04
120 min	-	0.19 ± 0.01	0.25 ± 0.06
References	[24]	This study	This study

**Table 2:** Concentration of loading solutions, drug loading (DL, mg/cm^3^), entrapment efficiency (EE, %), and Korsmeyer–Peppas model parameters obtained from the in vitro release kinetics of Na5_1_*^4^ hydrogels; R^2^, regression coefficient; *n*, release exponent.

API	Loading Solution (wt%)	DL (mg/cm^3^)	EE (%)	*n*	R^2^
Caffeine	1.0	11.98 ± 1.29	13.11 ± 0.49	0.523	0.996
Sodium Diclofenac	0.1	1.30 ± 0.05	14.70 ± 0.60	0.520	0.996
Salicylic Acid	0.1	1.49 ± 0.15	15.15 ± 1.54	0.666	0.996
Sodium Ibuprofen	1.0	11.25 ± 2.04	14.43 ± 0.88	0.491	0.995

## Data Availability

Data will be made available upon request.
